# Effect of Comorbid Diseases on Hospitalization and In-Hospital Mortality of Elderly Patients Who Reapplied to the Emergency Department Within 72 Hours

**DOI:** 10.7759/cureus.49558

**Published:** 2023-11-28

**Authors:** Muhammed Fuad Uslu, Mustafa Yilmaz, Metin Atescelik, Feti A Atilgan

**Affiliations:** 1 Department of Internal Medicine, Elazığ Fethi Sekin City Hospital, Elâzığ, TUR; 2 Department of Emergency Medicine, Firat University Faculty of Medicine, Elâzığ, TUR; 3 Department of Emergency Medicine, Elazığ Fethi Sekin City Hospital, Elâzığ, TUR

**Keywords:** unplanned revisit, revisit, multimorbidity, emergency medical service, elderly people

## Abstract

Objective: This study aimed to determine the effects of comorbidities on hospitalization and in-hospital mortality in patients aged 65 years and older who returned to the emergency department within 24, 48, and 72 hours of an initial visit.

Methods: This study was conducted at the Department of Emergency Medicine, Firat University Faculty of Medicine, Elâzığ, Turkey. It has a retrospective design and received local ethics committee approval from the university. Patients aged 65 years and older who presented to the emergency department within a one-year period (2022) were examined to identify those who returned to the emergency department within 24, 48, and 72 hours of an initial visit.

Results: A total of 763 (3.2%) patients >65 years of age returned to the emergency department within the first three days of their initial visit. Of these returning patients, 349 returned within 24 hours (Group 1), 227 within 48 hours (Group 2), and 187 within 72 hours (Group 3). Being female, polypharmacy, the presence of at least one comorbidity, cancer, and chronic renal failure were found to be independent predictors of hospitalization, whereas polypharmacy was found to be an independent predictor of in-hospital mortality.

Conclusion: Patients returning to the emergency department shortly after an initial visit should be assessed more diligently due to the risk of mortality.

## Introduction

With the increasing elderly population, the number of elderly patients visiting emergency departments (EDs) has been increasing [1]. Assessment of elderly patients presents various challenges compared with the assessment of younger patients due to comorbidities, polypharmacy, difficulty in expression due to cognitive problems, and the rapid evolution of their medical condition [2-5]. Elderly patients’ return to the ED shortly after an initial visit is prompted for various reasons, such as recurrence of symptoms or chronic illness, social and/or psychological characteristics, and/or low-quality care at the first visit [6-8]. Although some measures have been identified and different recommendations have been put forward to reduce the number of elderly patients returning to the ED, ED revisits remain a subject of research [9,10].

Patients returning to the ED within three days of an initial visit have to be assessed more carefully due to the possibility of detecting a disease that may have been missed during the initial visit or the chances of a clinical symptom that was previously considered non-specific becoming more specific [10].

Although previous studies have sought to identify the causes of short-term return visits to ED among elderly individuals, few have focused on assessing the impact of comorbidities [8,10,11]. According to studies, patients who frequently visit the ED are older, have more chronic medical conditions, are hospitalized more frequently, and are at a higher risk of death [2].

This study sought to analyze the effects of comorbidities on hospitalization and in-hospital mortality among patients aged 65 years and older who returned to the ED within one, two, and three days of an initial visit.

## Materials and methods

This study was conducted at the Department of Emergency Medicine, Firat University Faculty of Medicine, Elâzığ, Turkey. It has a retrospective design and received local ethics committee approval from the university. Records of all patients aged 65 years and over who presented to the ED within a one-year period were screened to identify those who were re-registered in the ED within 24, 48, and 72 hours of their initial visit. Age, gender, number of medications used, comorbidities, and mortality status of the patients were recorded in the data forms. Polypharmacy was defined as patients who regularly used five or more medications [12].

Statistical analysis

Study data were analyzed using the IBM SPSS Statistics for Windows version 21 (released 2012; IBM Corp., Armonk, New York, United States). Numerical data were expressed in mean ± standard deviation, and qualitative data were expressed in percentage. The distribution of continuous variables was tested using the Kolmogorov-Smirnov and Shapiro-Wilk normality tests. One-way analysis of variance was used to compare more than two independent groups. Categorical data were compared using the chi-square test (cross-tab). Binary logistic regression was used for dichotomous dependent variables. The enter method was used for the logistic regression analysis. For all analysis results, statistical significance was set at p < 0.05.

## Results

During the one-year period, 25,785 patients aged >65 years were found to visit the ED. Of these ED visits, 20,519 were single visits, whereas 5266 (20%) were repeat visits in different numbers and on different days. After being discharged from the ED, 763 (3.2%) patients >65 years of age returned to the ED within the first three days of their initial visit. Of these returning patients, 349 returned within 24 hours (Group 1), 227 within 48 hours (Group 2), and 187 within 72 hours (Group 3). Age (p = 0.075) and gender (p = 0.310) did not influence when they returned to the ED. The number of patients using five or more drugs (using polypharmacy) was found to be significantly less in patients who returned within Group 2 (p = 0.018). Analysis of comorbidities found no difference in the number of patients with at least one comorbidity (p = 0.742). Although there was a significant difference in the number of patients with cerebrovascular illness (p = 0.006), there was no significant difference in other comorbidities. In-hospital mortality was detected in 8.4% (n = 64) of the patients included in the study. The day of the return visit did not lead to a difference in the number of patients who died (p = 0.582) (Table 1).

**Table 1:** Characteristics of patients who returned to the emergency department within one, two, and three days of discharge. DM: diabetes mellitus, CAD: coronary artery disease, CVD: cerebrovascular disease, CRF: chronic renal failure

	Total	Group 1	Group 2	Group 3	p
N (F/M)	763 (403/360)	349 (174/175)	227 (127/100)	187 (102/85)	0.310
Age	73.9±7.13	73.1±6.51	74.6±7.44	74.5±7.72	0.075
Polypharmacy n(%)	109 (14.3)	59 (16.9)	20 (8.8)	30 (16.0)	0.018
Comorbidity n(%)	559 (73.3)	254 (45.4)	164 (29.3)	141(25.2)	0.742
Malignancy n(%)	97 (12.7)	40 (11.5)	33 (14.5)	24 (12.9)	0.563
Hypertension n(%)	327 (42.9)	141 (40.4)	92 (40.5)	94 (50.3)	0.062
DM n(%)	162 (21.2)	79 (22.6)	43 (18.9)	40 (21.4)	0.571
COPD n(%)	65 (8.5)	30 (8.6)	17 (7.5)	18 (9.6)	0.739
Heart failure n(%)	81 (10.6)	34 (9.7)	21 (9.3)	26 (13.9)	0.240
CAD n(%)	209 (27.4)	105 (30.1)	54 (23.8)	50 (26.7)	0.247
CVD n(%)	47 (6.2)	13 (3.7)	14 (6.2)	20 (10.7)	0.006
CRF n(%)	47 (6.2)	20 (5.7)	12 (5.3)	15 (8.0)	0.465
Alzheimer’s/dementia n(%)	26 (3.4)	14 (4.0)	7 (3.1)	5 (2.7)	0.682
Hospitalization n(%)	167 (21.9)	85 (24.4)	40 (17.6)	42 (22.5)	0.158
Mortality n(%)	64 (8.4)	33 (9.5)	18 (7.9)	13 (7.0)	0.582

Being female, polypharmacy, the presence of at least one comorbidity, cancer, and chronic renal failure were found to be independent predictors of hospitalization, whereas polypharmacy was found to be an independent predictor of in-hospital mortality (Table 2).

**Table 2:** Factors affecting hospitalization and in-hospital mortality COPD: chronic obstructive pulmonary disease, OR: odds ratio, CI: confidence interval

	Hospitalization			In-hospital mortality		
	OR	95% CI	p	OR	95% CI	p
Constant	0.026		0.001	0.417		0.664
Age	1.041	1.015-1.069	0.003	1.011	0.966-1.059	0.631
Being female	1.597	1.079-2.365	0.019	0.764	0.382-1.528	0.446
Polypharmacy	2.091	1.152-3.793	0.015	2.888	1.085-7.685	0.034
Comorbidity	4.741	2.197-10.230	<0.001	1.231	0.251-6.041	0.798
Malignancy	2.752	1.619-4.676	<0.001	2.006	0.834-4.827	0.120
Hypertension	0.758	0.487-1.180	0.220	0.729	0.323-1.645	0.446
Diabetes mellitus	1.313	0.820-2.100	0.256	0.567	0.234-1.374	0.209
COPD	1.049	0.565-1.949	0.879	1.277	0.441-3.692	0.652
Heart failure	0.961	0.534-1.731	0.895	1.075	0.404-2.859	0.885
Chronic artery disease	0.881	0.559-1.390	0.587	0.692	0.299-1.600	0.389
Cerebro-vascular disease	1.322	0.669-2.615	0.422	1.376	0.443-4.272	0.581
Chronic renal failure	2.906	1.505-5.614	0.001	1.487	0.540-4.093	0.443
Alzheimer/dementia	0.963	0.385-2.409	0.936	0.960	0.194-4.752	0.960
For hospitalization: R2 (Cox–Snell) = 0.151, R2 (Nagelkerke) = 0.232, p < 0.001. For in-hospital mortality: R2 (Cox–Snell) = 0.097, R2 (Nagelkerke) = 0.132, p = 0.003.						

## Discussion

This study found that the rate of patients aged >65 years who returned to the ED within the first 24, 48, and 72 hours of discharge after an initial visit was 3.2%. Among these patients, the in-hospital mortality rate was 8%. Polypharmacy, the presence of at least one chronic disease, malignancy, and chronic renal failure (CRF) were found to be independent predictors of hospitalization.

Riggs et al. [13] reported that, of 35,440 ED visits, 1,992 (5.62%) were repeat visits that happened within 72 hours of the initial visit and that patients frequently using the ED were more likely to return to the ED within the first 72 hours after discharge. This study found that 3.2% of patients >65 years of age returned to the ED within 72 hours of discharge.

Patients with chronic diseases use the ED repeatedly [14]. Chronic conditions (particularly mental disorders and respiratory diseases) and the presence of multiple chronic conditions, according to Di Bella et al. [15], were associated with repeated use of the ED. Similarly, Ko et al. [16] investigated the effect of comorbidities on repeat visits to the ED and reported that congestive heart failure, chronic obstructive pulmonary disease (COPD), peptic ulcer, liver disease, cancer, and psychiatric disorders increased the risk of frequent use of the ED. Moreover, Jones et al. [5] followed elderly individuals aged 66 years and older with dementia over a one-year period and reported that two-thirds (66.1%) visited the ED at least once, 39.4% at least twice, and 23.5% three or more times during the year. However, Gruneir et al. [17] reported that nursing home residents with Alzheimer’s disease or dementia and with five or more chronic diseases were less likely to be frequent users of the ED. This study found that the presence of at least one comorbidity, cancer, and CRF were independent predictors of hospitalization among patients who returned to the ED within the first three days after discharge and were hospitalized at a repeat visit.

As life expectancy increases, individuals are more likely to experience a greater number of health problems and to be treated with multiple medications. Although polypharmacy has beneficial effects in terms of reducing mortality and alleviating symptoms among elderly patients with multimorbidity, it also leads to certain risks linked to the complexity of the different types of drugs, the risk of drug-drug interactions, and the risk of drug-disease interactions [18,19]. Polypharmacy also affects the hospitalization of patients. Polypharmacy at the time of admission was found to be an independent risk factor for prolonged hospitalization in patients undergoing gastrointestinal surgery in one study [20]. Chang et al. [21] discovered that hospitalized geriatric patients were 18% more likely to be readmitted within five years. The present study found polypharmacy to be an independent predictor of hospitalization.

Studies that investigated polypharmacy and mortality have reported conflicting results. Polypharmacy was linked to a twofold increase in the risk of non-cancer death in adults; Turgeon et al. [22] and Lu et al. [23], on the other hand, did not find an association between polypharmacy and mortality risk among adults aged 65 years and older. The present study found that polypharmacy was an independent predictor of in-hospital mortality.

Limitations

This study has some limitations. First, the study was retrospective and based on data available in patient files, and information on previous surgeries and angiography was obtained from single-center records and verbal reports of patients and/or their caregivers. Second, the study did not divide patients into trauma and non-trauma groups, did not examine them for vital signs and diagnoses at the time of admission to the hospital, and reviewed only in-hospital mortality but did not include data on out-of-hospital mortality. These limitations should be addressed in future studies.

## Conclusions

Being female, the presence of at least one comorbidity (cancer and CRF), and polypharmacy were found to be independent risk factors for hospitalization of patients aged 65 years and older at their first repeat visit after an initial discharge from the ED. Polypharmacy was also found to be an independent predictor of in-hospital mortality among these patients. Elderly patients should be treated as special patients in emergency departments. Although it is not common for this patient group to be re-admitted in a short time, more care should be taken when making the decision to discharge from the emergency department, especially in patients with cancer and CRF.
